# Patient-reported outcomes after upper-extremity deep vein thrombosis due to compression syndromes: a multinational longitudinal retrospective study

**DOI:** 10.1016/j.rpth.2026.103358

**Published:** 2026-01-16

**Authors:** Maya Abrishami Kashani, Domenico Baccellieri, Mert Dumantepe, Naomi Elbaranes, Nicolas Gendron, Marianna Gigliotti De Fazio, Egidio Imbalzano, Lina Khider, Nils Kucher, Corrado Lodigiani, Benedetta Madaro, Saskia Middeldorp, Clara Sacco, Yasmine Terbeche, Ferdinando Benito Attilio Valente, Stefano Barco

**Affiliations:** 1 Department of Angiology, University Hospital Zurich, Zurich, Switzerland; 2 Vascular Surgery Department, IRCCS H. San Raffaele, Milan, Italy; 3 Department of Cardiovascular Surgery, Uskudar University School of Medicine, Istanbul, Turkey; 4 Hematology Department, Hôpital Européen Georges Pompidou, Assistance Publique – Hôpitaux de Paris-Centre Université Paris Cité (APHP-CUP), F-75015 Paris, France; 5 Paris Cité University, INSERM, Paris Cardiovascular Research Centre, Team Endotheliopathy and Hemostasis Disorders, Paris, France; 6 F-CRIN INNOVTE, F-42000 Saint-Étienne, France; 7 Department of Clinical and Experimental Medicine, University of Messina, Messina, Italy; 8 Vascular Medicine Department, Hôpital Européen Georges Pompidou, Assistance Publique – Hôpitaux de Paris-Centre Université Paris Cité (APHP-CUP), Paris, France; 9 IRCCS Humanitas Research Hospital, Center for Thrombosis and Hemorrhagic Diseases; 10 Internal Medicine Residency Program, School of Medicine, University of Insubria, Varese and Como, Italy, Varese; 11 Department of Internal Medicine, Radboud university medical center, Nijmegen, Netherlands; 12 Center for Thrombosis and Hemostasis, Johannes Gutenberg University Mainz, Mainz, Germany

**Keywords:** outcome assessment (health care), quality of life, postthrombotic syndrome, upper-extremity deep vein thrombosis, venous thromboembolism

## Abstract

**Background:**

There are limited data on functional and patient-reported outcomes after acute upper-extremity deep vein thrombosis (UEDVT) caused by compression syndromes.

**Objectives:**

This study examined patient-reported functional impairment, quality of life, and treatment satisfaction after UEDVT.

**Methods:**

We did a retrospective multicenter cohort study of patients with confirmed UEDVT due to compression syndromes not related to central lines. Patients were included at 5 centers in 4 countries and completed a standardized patient-reported outcome survey. The primary outcome was upper-limb function measured via the Quick Disabilities of the Arm, Shoulder, and Hand (QuickDASH) score (range, 0-100; higher scores indicate worse function) during follow-up. Quality of life was assessed using a Visual Analogue Scale. Additional outcomes included persistent symptoms, treatment satisfaction, mental health impact, and perception of unmet needs.

**Results:**

We included 82 patients (median age, 38 years; Q1-Q3, 26-48 years); 44% were women. All patients received anticoagulation, and 66% underwent catheter-directed therapy. First-rib resection was performed in 24% of patients. After a median follow-up of 16 months, 63% reported ≥1 persistent symptom, most commonly swelling (30%) and heaviness (29%). The median QuickDASH score was 8 (Q1-Q3, 2-20), indicating mild overall disability. QuickDASH correlated negatively with quality of life (Spearman ρ = −0.82; *P* < .001) and across QuickDASH items and countries. Symptom persistence emerged as the main predictor of residual functional impairment. Patient-reported priorities included maintaining adequate arm function (78%) and avoiding recurrent UEDVT (73%).

**Conclusion:**

UEDVT can have lasting functional and psychosocial effects. Patient-reported outcomes reveal a burden that is not captured by traditional clinical metrics and should be integrated into routine care as well in the setting of interventional trials.

## Essentials

• Upper-extremity deep vein thrombosis (UEDVT) affects healthy, active patients.
• Patients from 5 hospitals in 4 countries completed a standardized outcomes survey.
• Nearly two-thirds had lasting arm symptoms, mainly swelling and heaviness.
• Ongoing symptoms were linked to worse arm function and quality of life.

## 1 Introduction

Upper-extremity deep vein thrombosis (UEDVT) accounts for 5% to 7% of all venous thromboembolic events [[1], [2], [3]]. It is typically caused by local triggers, such as central venous lines and active cancer. In 10% to 20% of cases, however, UEDVT affects younger, active individuals after strenuous upper-limb activity. This form, also known as effort-related UEDVT or Paget-Schroetter syndrome, is usually triggered by a chronic compression of the subclavian vein between the first rib and clavicle (thoracic inlet syndrome) due to physical activity or anatomical variations [4].

Typical symptoms of acute UEDVT include swelling, pain, a sensation of tightness, and functional impairment of the affected arm. Anticoagulation is the mainstay of treatment. A significant residual compression at the level of the thoracic inlet syndrome may cause residual symptoms and thrombosis recurrence [5,6]. In selected patients, endovascular reperfusion and first rib resection are considered [7,8], although data remain limited.

Most studies focus on radiological outcomes such as venous patency or reintervention. However, these do not capture the functional and psychosocial burden experienced by patients. Preserving upper-extremity (UE) function and health-related quality of life (QoL) is a key goal in patients with effort-related UEDVT, who are often young, physically active individuals. Persistent pain, swelling, and functional limitation may impair return to work, sports, and daily activities. In contrast to lower-limb deep vein thrombosis (DVT), for which validated patient-reported outcome measures and postthrombotic syndrome (PTS) scores exist, no widely accepted tools or standardized outcomes have been developed for UEDVT. This retrospective, longitudinal, multicenter, multinational study aimed to fill this gap by evaluating patient-reported outcomes in patients with a history of UEDVT using standardized tools including the QuickDASH and a QoL scale.

## 2 Methods

### 2.1 Patients

This longitudinal cohort study was conducted at 5 vascular care centers in Switzerland, Italy, France, and Turkey. We included patients with objectively-confirmed UEDVT that was considered to be related to a local anatomical compression based on clinical assessment supported by at least 1 of the following: (i) procedural findings during catheter-directed treatment (eg, thrombectomy and/or angioplasty), suggesting venous obstruction at the thoracic outlet; (ii) duplex ultrasound demonstrating residual stenosis or occlusion in the thoracic outlet region; or (iii) functional venous imaging (computed tomography or magnetic resonance venography) consistent with dynamic compression. Patients with UEDVT secondary to central venous catheters or lines were excluded.

Consecutive patients were identified by searching institutional databases for confirmed UEDVT cases between 2016 and 2025. A standardized patient-reported outcome questionnaire—originally developed and in use at the coordinating center in Zurich for routine clinical care—was translated and distributed to all sites and administered by trained personnel, either in person or remotely. For the QuickDASH score, versions in different languages were available. Patients were asked to complete the questionnaire either during scheduled follow-up visits as part of routine care or, if no clinical visit was planned, remotely via phone or email. Clinical data, including treatment type and timing, were retrospectively extracted from electronic health records in the occasion of the latest contact with the patient. Treatment strategies reflected real-world clinical practice at individual centers and may include anticoagulation alone or reperfusion treatments, such as catheter-directed thrombolysis, followed by anticoagulation. Data on first rib resection during follow-up were collected. Ethical approval and written informed consent forms were obtained at each site according to national requirements.

### 2.2 Outcome measures

The primary outcome was upper-limb function, measured using the QuickDASH questionnaire. This tool was recommended for assessing functional outcomes after UEDVT in a previously published Delphi consensus [9]. QuickDASH includes 11 questions about arm, shoulder, and hand function. Each item is rated from 0 (no difficulty) to 4 (unable to perform). The QuickDASH score is calculated as the sum of item scores divided by the number of completed items × 25. The final score ranges from 0 to 100, where higher values indicate greater disability. To evaluate overall subjective health status, the QuickDASH score was compared with a Visual Analogue Scale (VAS). The VAS captures patients’ perceived QoL on a scale from 0 to 100, with 100 representing the best QoL.

We did not quantify the different severity grades of the UE-PTS score [9,10] as it lacks harmonization and standardization, and its assessment would have not been possible during phone interviews, as some parameters, such as arm diameter, discoloration, and presence of varicose veins, should have been objectively measured or observed during an in-person visit.

Patients were asked whether UEDVT impaired their mental health and to which extent and which UEDVT-specific fears emerged during follow-up. In addition, we studied the persistence of symptoms, treatment-related complications (eg, bleeding and recurrent thrombosis), and treatment satisfaction. Patients were also asked to share their preferences concerning the treatment they had received and what they feel it should be considered a priority to monitor the course of acute treatment. The complete list of questions is available as Supplementary Material.

The safety outcome was the incidence of bleeding events during the period of anticoagulant therapy, according to the International Society on Thrombosis and Haemostasis (ISTH) classification. A major bleeding was defined by a fatal bleeding and/or symptomatic bleeding in a critical area or organ, such as intracranial, intraspinal, intraocular, retroperitoneal, intra-articular or pericardial, or intramuscular with compartment syndrome; bleeding causing a fall in hemoglobin levels of 20 g/L or more; or leading to a transfusion of 2 units or more of whole blood or red cells [11]. A clinically relevant nonmajor bleeding (CRNMB) was defined as a bleeding not falling under the definition of major bleeding but either requiring medical intervention by a health care professional or leading to increased level of care [12]. A minor bleeding corresponded to any bleeding not classified as major or CRNMB. In our cohort, patients with multiple bleedings were classified according to their worst bleeding event. Data on the exact type and dosing of anticoagulant therapy were incomplete and therefore could not be systematically analyzed; however, all patients received therapeutic anticoagulation in accordance with local and contemporary clinical standards.

### 2.3 Statistical analysis

Descriptive statistics were used to summarize patient characteristics and outcomes. Categorical variables were reported as frequencies and percentages and continuous variables as means with SDs or medians with IQRs. To explore the relationship between QuickDASH score and QoL, we calculated Spearman correlation. QuickDASH scores were also grouped into 5 categories (minimal, 0-20; mild, 21-40; moderate, 41-60; severe, 61-80; and very severe, 81-100), and group differences in QoL were tested using the Kruskal–Wallis test. Group comparisons, including those between the 2 predefined management strategies (reperfusion treatment vs anticoagulation alone), were performed using chi-squared tests for categorical variables and *t*-tests or Mann–Whitney U-test for continuous variables. Missing data were handled using complete-case analysis. All analyses were performed using the R software (R Foundation for Statistical Computing) and JASP software (JASP), with 95% CIs and *P* values of <.05 considered statistically significant.

## 3 Results

### 3.1 Demographic and clinical characteristics

A total of 82 patients completed the follow-up survey at 5 centers. The median age at UEDVT diagnosis was 38 years (Q1-Q3, 26–48 years), and 44% were women (Table 1). Most patients (77%) had UEDVT associated with anatomical thoracic inlet compression, whereas the remaining patients had secondary reasons of possible compression including an atypical local compression. A family history of VTE was described in 17 (21%) patients, and 11 (13%) patients had had a prior VTE. Anticoagulation therapy was prescribed to all patients, and the anticoagulant treatment was taken over a median time of 270 days (Q1-Q3, 134-547 days) (Table 2). Catheter-directed treatment followed by anticoagulation was performed in 54 (66%) patients, and 28 (44%) of patients received anticoagulation alone. Compression stockings were used in approximately 50% of patients after UEDVT diagnosis. Patients were followed up over a median in-person clinical follow-up of 12 months (Q1-Q3, 6-29 months), whereas interviews were delivered during follow-up visits after a median of 16 months (Q1-Q3, 4–39 months). During follow-up, 20 (24%) patients underwent first rib resection. A total of 17 (21%) patients experienced a recurrent UEDVT at any time during follow-up. A recurrent UEDVT event was documented in 24.1% of patients treated interventionally (13/54) compared with 10.7% of those treated conservatively (3/28). This difference was not statistically significant (Fisher exact test, *P* = .24). One major and 3 CRNMBs occurred during anticoagulation.

**Table 1:** Baseline and demographic characteristics of patients with UEDVT.

Characteristic	Values (*N* = 82)
Demographic	
Age at index UEDVT (y)	38 (26-48)
Women	36 (44.0)
Body mass index (kg/m^2^)	24.0 (22.0-27.5)
Risk factors for VTE	
No known comorbidity	36 (44.0)
Known malignancy (active or under treatment)	7 (8.5)
Estrogen-based contraceptives or pregnancy	8 (9.8)
Prior VTE	11 (13.0)
Family history of VTE	17 (21.0)
Phenotype 1: Paget-von-Schroetter syndrome	63 (77.0)
Phenotype 2: other compression or chronic disorder excluding central lines	17 (21.0)
Both	2 (2.4)
Associated risk factors	
Compression at anatomical thoracic inlet	58 (71.0)
Anatomical narrowing	51 (62.0)
Trauma or injury	9 (11.0)
Repeated mechanical stress	35 (43.0)
Thrombophilia (any)	22 (27.0)
Long-haul travel	6 (7.3.0)
Iatrogenic	9 (11.0)
Known cardiovascular disease	14 (17.0)
Thrombus location	
Brachiocephalic vein or superior vena cava	10 (12.0)
Subclavian vein	76 (93.0)
Axillary vein	51 (62.0)
Brachial vein	22 (27.0)
Forearm veins	3 (3.7)
Superficial veins^a^	7 (8.6)

Values are *n* (%) or median (Q1-Q3).

UEDVT, upper extremity deep vein thrombosis; VTE, venous thromboembolism.

a Concomitant superficial vein thrombosis was defined as thrombosis of a superficial upper-extremity vein documented at index assessment for acute UEDVT.

**Table 2:** Treatment of UEDVT and risk of adverse events.

Adverse events	Value (*N* = 82)
Treatment of UEDVT	
Length of in-hospital stay (d)	2 (1-5)
Duration of anticoagulation (d)	270 (134-547)
Type of invasive intervention in the acute phase	
Catheter-directed thrombolysis	36 (44.0)
Percutaneous thrombectomy	35 (43.0)
Balloon angioplasty	43 (52.0)
Surgical thrombectomy followed by rib resection	3 (3.7)
Use of compression stockings after UEDVT	37 (47.0)
Rib resection during follow-up	20 (24.0)
Risk of adverse events	
Time of latest in-person follow-up visit (mo)	12 (6-29)
Recurrent UEDVT	17 (21.0)
Bleeding events (ISTH criteria)	17 (21.0)
Minor bleeding	14 (19.0)
Clinically relevant nonmajor bleeding	3 (4.2)
Major bleeding	1 (1.0)

Values are *n* (%) or median (Q1-Q3).

ISTH, International Society on Thrombosis and Haemostasis; UEDVT, upper extremity deep vein thrombosis.

### 3.2 Assessment of functional status and patient-reported outcomes

**Figure 1:**
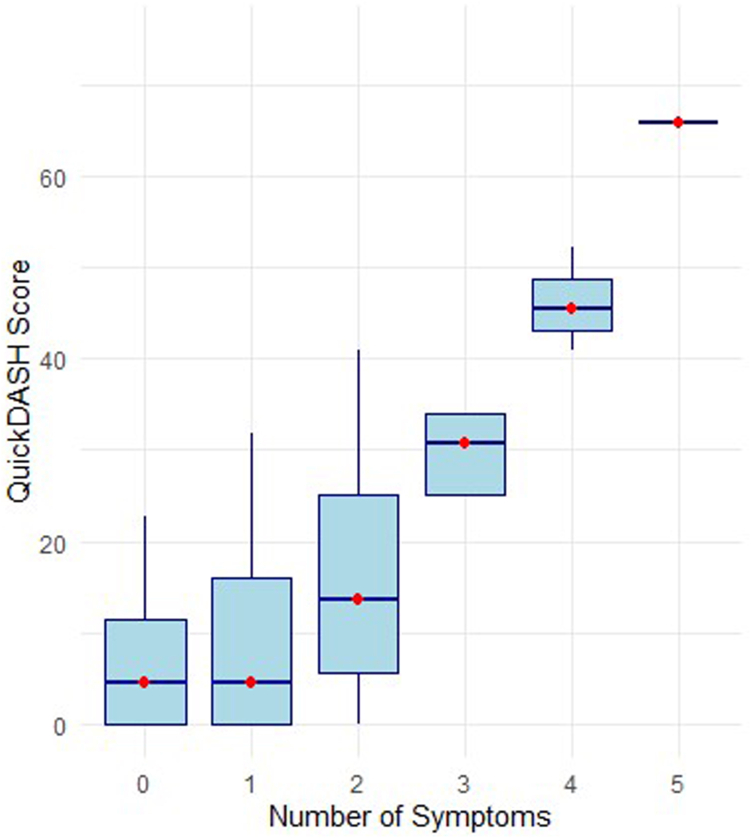
Median QuickDASH score (y axis) across groups of patients stratified according with the number of symptoms of post-thrombotic syndrome (x axis) during follow-up. Blue boxplots depict the distribution (Q1-Q3) of patient-reported upper-extremity function according to the QuickDASH score, and red dots indicate the median values. QuickDASH, Quick Disabilities of the Arm, Shoulder, and Hand.

A total of 51 (62%) patients had at least 1 residual symptom at the time of functional assessment after UEDVT, 32 (39%) had at least 2 residual symptoms, and 18 (22%) had 3 or more residual symptoms (Table 3). The most frequent ones were represented by swelling (30%), heaviness (29%), and pain (16%). QuickDASH scores increased with the number of residual symptoms, with higher symptom counts associated with greater functional impairment. Median QuickDASH scores ranged from 2.3 in patients with 1 symptom to 65.9 in those reporting 5 symptoms, as illustrated in Figure 1.

**Table 3:** Patient-reported outcomes on functional impairment and psychosocial impact after UEDVT.

Outcomes	Value (*N* = 82)
Time of functional assessment since diagnosis (mo)	16 (4-39)
At least one residual symptom	52 (63.0)
Swelling	25 (30.0)
Pain	13 (16.0)
Heaviness	24 (29.0)
Reduced mobility	7 (8.5)
Skin discoloration	7 (8.5)
Visible veins	24 (29.0)
Paresthesia	7 (8.5)
Weakness	3 (3.7)
Discomfort	4 (4.9)
QuickDASH score	8 (2-20)
QoL score	8.00 (7.00-9.50)
Currently receiving anticoagulation therapy	34 (41.0)
Condition affected mental health	
No	41 (50.0)
Yes, mildly	30 (37.0)
Yes, severely	11 (13.0)
If yes, what effects have you noticed?	
Fear of thrombosis	33 (40.0)
Fear of bleeding	9 (11.0)
Restricted social activities	17 (21.0)
Sleep problems	11 (13.0)
Depression	1 (1.2)
Burnout	1 (1.2)
Received psychological support (eg, counseling) due to persisting venous thoracic inlet compression: answers to question	
“No, but I would have liked it”	9 (11.0)
“No, it was not necessary”	58 (73.0)
“Yes”	13 (16.0)

QoL, quality of life; QuickDASH, Quick Disabilities of the Arm, Shoulder, and Hand.

**Figure 2:**
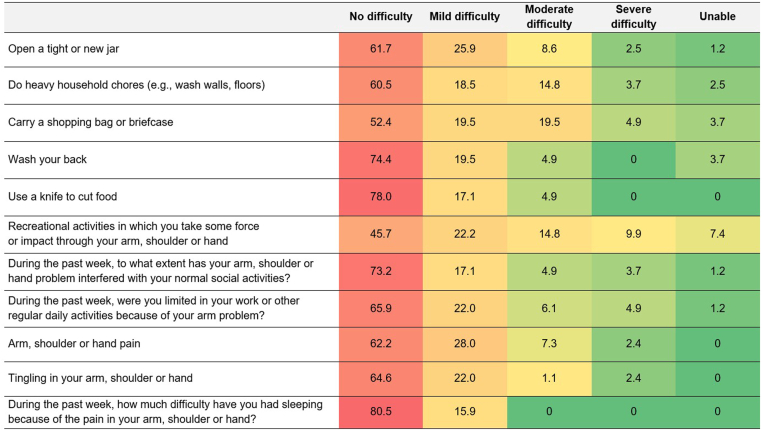
Individual components of the QuickDASH score. Numbers indicate the percentage of patients presenting with each individual item out of total patients in the row. QuickDASH, Quick Disabilities of the Arm, Shoulder, and Hand.

The median QuickDASH score was 8 points (Q1-Q3, 2–20 points) of a maximum of 100 points (100 corresponds to the worst functional performance). The 11 functional components of the QuickDASH score are displayed in Figure 2. The median (Q1-Q3) values of QuickDASH score across countries were similar and are displayed in Supplementary Table 1.

A total of 91% of respondents reported being either satisfied or very satisfied with their treatment. However, patients reported that UEDVT affected their mental health severely in 13% (*n* = 11) of cases and mildly in 37% (*n* = 30) of cases, particularly concerning the fear of a recurrent thrombosis (*n* = 33, 40%), limited social activities (*n* = 17, 21%), sleep problems (*n* = 11, 13%), or fear of bleeding (*n* = 9, 11%). When asked whether they had received psychological support (eg, counseling) in the context of their condition, most respondents (*n* = 58, 72.5%) stated that it had not been necessary. However, 9 patients (11.3%) reported that they had not received support but would have liked to, and 13 patients (16.3%) confirmed that they had received psychological support.

### 3.3 Predictors of poorer functional status and correlation between different scales

**Figure 3:**
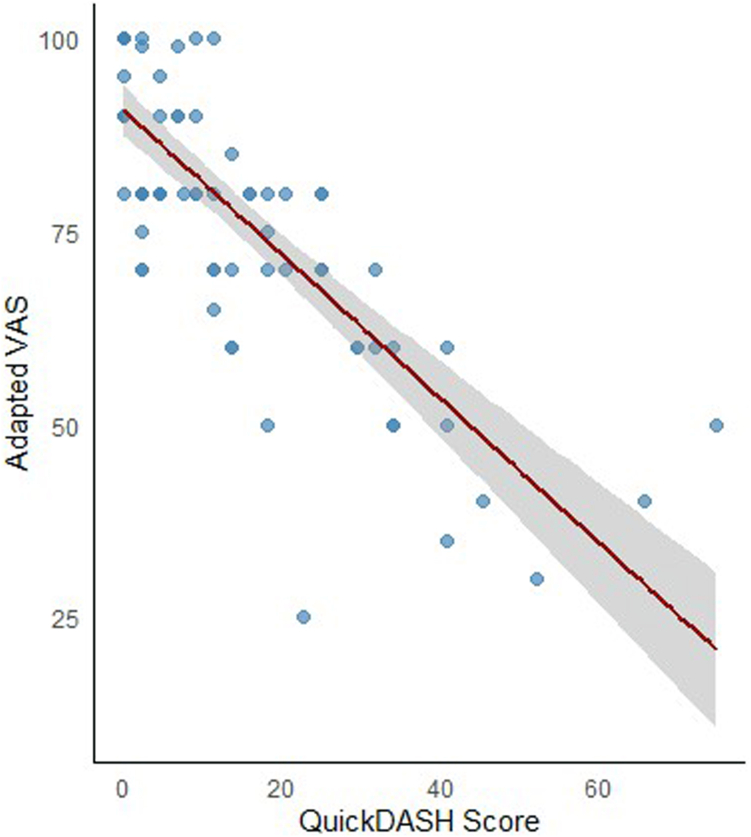
Correlation between the QuickDASH score and the adapted VAS for quality of life. QuickDASH, Quick Disabilities of the Arm, Shoulder, and Hand; VAS, Visual Analogue Scale.

As reported in Table 4, the presence of symptoms during follow-up emerged as the main predictor of poorer functional status according to the QuickDASH score (β, 8.7 points for the presence of symptoms; 95% CI, 1.7-15.7) after adjusting for age, sex, time since diagnosis, and type of intervention. In a multivariable exploratory analysis (Supplementary Table 2), we studied the association between individual symptoms and QuickDASH. Limited mobility, skin discoloration, and arm weakness appeared to have the highest impact on functional limitations, followed by paresthesia and swelling. The median adapted VAS for QoL was 8 points (Q1-Q3, 7.0-9.5 points) of a maximum of 10 points (10 corresponded to the best QoL according to the adapted VAS). We observed a strong negative correlation between QuickDASH and QoL (Spearman ρ = –0.82; 95% CI, −0.99 to −0.74; *P* < .001), suggesting that patients with greater disability reported lower adapted VAS for QoL (Figure 3). We confirmed an existing correlation between QuickDASH score and QoL across the 11 components of the QuickDASH score, as depicted in Supplementary Table 3 and Supplementary Figure 1. The correlation between QuickDASH score and QoL across countries was confirmed and is depicted in Supplementary Figure 2. To evaluate whether variability in follow-up timing influenced patient-reported outcomes, patients were stratified according to the interval between UEDVT diagnosis and completion of the questionnaire (<12 vs ≥12 months). Median follow-up was 7.1 months (IQR, 5.2-10.0 months) in the <12-month group (*n* = 37) and 19.4 months (IQR, 14.1–27.3) in the ≥12-month group (*n* = 45). The median UE function score (QuickDASH) tended to be higher among patients assessed earlier (13.6 [IQR, 27.3] vs 4.5 [IQR, 11.4]), indicating poorer functional outcomes, although this difference did not reach statistical significance (*P* = .06). Median QoL scores were similar between groups (8.0 [IQR, 3.0] vs 8.0 [IQR, 2.9]; *P* = .12).

**Table 4:** Univariate and multivariable linear regression model for the predictors of QuickDASH score.

Variables	Univariate analysis, β (95% CI)	Multivariable analysis, β (95% CI)
Age (y, per unit increase)	0.3 (0.1-0.5)	0.2 (0.1-0.45)
Female (vs male) sex	2.4 (−4.6 to 9.4)	1.4 (−5.4 to 8.1)
Time since diagnosis (mo, per unit increase)	−0.1 (−0.2 to 0.1)	−0.1 (−0.2 to 0.1)
Persistent symptoms (vs no symptoms)	9.9 (2.9-16.8)	8.7 (1.7-15.7)
Percutaneous thrombectomy (vs no thrombectomy)	−3.8 (−10.9 to 3.2)	−4.3 (−11.7 to 3.0)
Local thrombolysis (vs no thrombolysis)	−2.7 (−9.7 to 4.3)	−0.3 (−7.8 to 7.3)
Rib resection (vs no rib resection)	3.1 (−5.1 to 11.4)	3.0 (−4.9 to 10.9)

QuickDASH, Quick Disabilities of the Arm, Shoulder, and Hand.

Finally, we studied the potential correlation between the QuickDASH score and mental health (Supplementary Figure 3). The median QuickDASH score was 6.8 (Q1-Q3, 2.2-15.9) among participants without mental health issues (*n* = 1), 8.3 (Q1-Q3, 0.6-22.2) in those mildly affected (*n* = 30), and 13.6 (Q1-Q3, 3.4-40.9) in those reporting a higher burden of mental health issues (*n* = 11; *P* = .049.

### 3.4 Patients’ values and expectations

Patients reported that the most frequently cited goals were maintaining arm function (78%) and preventing recurrence (73%), followed by symptom relief (50%) and minimizing bleeding risks (29%) (Supplementary Table 4). When asked about unmet needs, 37% of patients reported a desire for greater support in coping with the consequences of their condition, and 11% expressed a wish for better explanation of their diagnosis and treatment options. Only a minority had received psychological support, although 11% indicated they would have welcomed it.

## 4 Discussion

This multinational, longitudinal study provides one of the few analyses of patient-reported outcomes after UEDVT, notably for effort-related forms associated with thoracic inlet compression. While most of patients with prior UEDVT included in this study reported mild disability and high satisfaction with care, our findings reveal a considerable burden of persistent symptoms, reduced QoL, and mental health strain that is not captured by traditional clinical outcomes alone, such as vessel patency and recurrent DVT, or by scales for PTS [13,14]. These findings indicate that functional end points should be assessed in clinical practice and be integrated in future trials on UEDVT to assess the impact on patients’ daily lives, particularly because this patient group is usually young and otherwise healthy and physically active.

In our study, we showed that >60% of patients with UEDVT had residual symptoms after >1 year from the acute event, usually consisting of swelling, heaviness, and pain. Although the overall functional status according to the QuickDASH was only mildly impaired, namely most of patients could perform daily activities without limitations, we found a clear correlation between residual symptoms and patients’ functionality, as well as with reported QoL and mental status. In this context, we support the concept of routinely assessing different functional domains after UEDVT (and other vascular conditions) and include these in future interventional studies. Of note, 16.3% of patients received psychological support after UEDVT. Consistently, a substantial proportion of patients reported mental health concerns, including fear of recurrence, sleep problems, and social limitations. These findings are consistent with recent literature on the psychological burden of DVT and pulmonary embolism [15]. In line with this, 37% of respondents indicated a preference for more support in coping with the consequences of their condition, also including functional rehabilitation and psychological support.

The QuickDASH score showed a strong inverse correlation with QoL, supporting its construct validity as a tool for assessing functional recovery after UEDVT. This is consistent with recent Delphi-based recommendations endorsing QuickDASH for upper-limb outcome assessment in thrombosis research. In our study, we provided initial validation of QuickDASH across countries (and different languages) and after comparison with scales assessing the general QoL and the mental status. Still, no universally accepted UE-PTS scoring system exists that can encompass signs and symptoms of PTS, similar to the Villalta scale for lower-extremity DVT, and patient-reported outcomes [10].

The current management of effort-related UEDVT remains controversial. Anticoagulation and compression stockings are the mainstay, extrapolated from evidence on the treatment of lower limb DVT. However, the use of direct oral anticoagulants (DOACs) after UEDVT is still debated. Although DOACs are standard of care for the treatment of acute DVT and pulmonary embolism and they may be effective and safe for UEDVT [16], they are not currently approved in many countries for this indication. Furthermore, there is lack of evidence on the duration of anticoagulation and the potential benefits of secondary prophylaxis with DOACs at a reduced dose. It is unclear whether anticoagulation may improve functional outcomes by preventing recurrent UEDVT and whether the rates of recurrence in patients on anticoagulation are comparable with those in other treatment groups of patients with VTE.

Another aspect that remains heavily debated concerns the invasive management of UEDVT, encompassing catheter-directed thrombectomy or local thrombolysis for the treatment of acute UEDVT, and the necessity, timing and choice of decompression treatments, including venoplasty and rib resection, during follow-up [17]. As outlined in a 2011 review [18], catheter-directed thrombolysis is indicated for recent, extensive UEDVT with severe swelling or impairment, while mechanical thrombectomy should be reserved for refractory cases in centers with expertise. Surgical decompression of the thoracic inlet may help reduce the rate of PTS, but evidence is limited as no randomized trials have compared interventional and conservative strategies to date. As UEDVT due to compression syndromes may arise from repeated mechanical compression of the subclavian vein or pre-existing anatomical variations, the persistence of symptoms may be related not only to an incomplete resolution of acute clots but also to the persistence of a chronic, in some cases pre-existing, venous compression and functional occlusion. To some extent, this condition is like what is observed in iliofemoral DVT and PTS due to May-Thurner anatomy, for which endovascular therapies may lead to an improvement of functional outcomes [19,20]. Although, in our study, we observed a tendency toward better functional outcomes following early catheter-directed thrombectomy and current guidelines suggest endovascular reperfusion in case of severe symptoms [17], more evidence is needed to support this statement.

This study highlights unmet needs in the postacute care of patients with UEDVT [15]. Patients may be unsure concerning the risk of developing functional limitations and recurrent UEDVT [21]. Several patients expressed a desire for more individualized treatment planning and clearer explanations of their diagnosis and prognosis. This may be since this information cannot be actively transmitted by the physician due to the lack of firm estimates and validated strategies. This highlights the need for shared decision making and improved communication, especially in a condition like UEDVT where evidence is lacking. Future prospective studies may help in providing more reliable estimates and, at the same time, introduce patients’ choice as one of the criteria to offer bailout (invasive) treatment strategies.

Limitations must be acknowledged. First, the retrospective and observational design introduces the risk of selection and recall bias. Patients who experienced persistent problems may have been more likely to participate in follow-up, while those who recovered fully may have been underrepresented, or vice versa. Recurrent events tended to be more common after interventional therapy than with anticoagulation alone. This difference was not statistically significant. As the treatment allocation was nonrandomized, one cannot exclude that more severe cases underwent intervention. Importantly, recurrent events are differently assessed: in the group of patients undergoing intervention, these should be interpreted as new-onset thrombosis in a vein that was functional after thrombectomy and venoplasty. In the group of patient treated with anticoagulation alone, a restoration of the distal subclavian vein is not expected due to the postthrombotic changes of vessel wall and chronic functional occlusion: in this context, recurrent events should be interpreted as thrombus extension toward more proximal segments or into collateral veins. These exploratory findings should therefore be interpreted cautiously. Additionally, symptom duration before diagnosis and treatment was inconsistently documented and therefore not available for analysis, which limits the assessment of the relationship between symptom onset, diagnostic delay, and early patient-reported outcomes. Follow-up intervals also varied among patients, reflecting the retrospective and multicenter design of this study. In a post hoc subgroup analysis stratified according to the length of follow-up (<12 vs ≥12 months), UE function and QoL scores were similar. We acknowledge that temporal heterogeneity may reduce the precision with which outcomes can be linked to a specific phase of recovery after UEDVT. Similarly, we did not collect information on not eligible patients, such as those with catheter-related thrombosis, and we did not systematically collect data on the specific type, dosage, and duration of anticoagulant therapy. We do not expect major differences between anticoagulants [[22], [23], [24]], but the length of anticoagulation may have played a role on the risk of recurrence and the indication for rib resection. Future prospective studies with standardized treatment documentation are warranted to investigate whether specific anticoagulation strategies influence long-term functional outcomes. Furthermore, we did not assess patient handedness, although this may influence the degree of functional impairment and, consequently, the impact on QoL. Prior literature showed that UEDVT due to thoracic inlet syndrome usually affects the dominant arm. Finally, we assessed health-related QoL using an adapted single-item VAS rather than a multidimensional, validated QoL instrument. This represents a pragmatic simplification chosen to facilitate implementation across centers and languages in a retrospective cohort. While the VAS captures patients’ global symptom burden, it may not fully reflect broader domains of QoL such as mental health, social participation, or work productivity. Future prospective studies should incorporate comprehensive, validated QoL measures to better characterize the long-term impact of compression-related UEDVT.

In conclusion, our findings indicate that the functional status was only mildly impaired after acute UEDVT caused by compression syndromes. Residual symptoms and psychological concerns were common after the acute phase and correlated with functional outcomes, as measured by the QuickDASH score. A standardized use of functional scales and patient-reported outcomes should be integrated in everyday clinical practice and in the setting of future interventional studies.

## Declarative of generative AI and AI-assisted technologies in the writing process

During the preparation of this work, the authors used ChatGPT with moderation in order to improve readability and language. After using this tool, the authors reviewed and edited the content as needed and take full responsibility for the content of the publication.

## References

[1] Ageno W., Haas S., Weitz J.I., Goldhaber S.Z., Turpie A.G.G., Goto S. (2019). Upper extremity DVT versus lower extremity DVT: perspectives from the GARFIELD-VTE Registry. Thromb Haemost.

[2] Isma N., Svensson P.J., Gottsäter A., Lindblad B. (2010). Upper extremity deep venous thrombosis in the population-based Malmö thrombophilia study (MATS). Epidemiology, risk factors, recurrence risk, and mortality. Thromb Res.

[3] Cote L.P., Greenberg S., Caprini J.A., Tafur A., Choi C., Muñoz F.J. (2017). Comparisons between upper and lower extremity deep vein thrombosis: a review of the RIETE Registry. Clin Appl Thromb Hemost.

[4] Khan O., Marmaro A., Cohen D.A. (2021). A review of upper extremity deep vein thrombosis. Postgrad Med.

[5] Urschel H.C., Patel A.N. (2008). Surgery remains the most effective treatment for Paget-Schroetter syndrome: 50 years’ experience. Ann Thorac Surg.

[6] Héron E., Lozinguez O., Emmerich J., Laurian C., Fiessinger J.N. (1999). Long-term sequelae of spontaneous axillary-subclavian venous thrombosis. Ann Intern Med.

[7] Panther E.J., Reintgen C.D., Cueto R.J., Hao K.A., Chim H., King J.J. (2022). Thoracic outlet syndrome: a review. J Shoulder Elbow Surg.

[8] Ohman J.W., Thompson R.W. (2020). Thoracic outlet syndrome in the overhead athlete: diagnosis and treatment recommendations. Curr Rev Musculoskelet Med.

[9] Schropp L., Cats R.B., de Kleijn R., van Hattum E.S., Middeldorp S., Nijkeuter M. (2023). The upper extremity postthrombotic syndrome score: an international Delphi consensus study to determine the score's functional disability component. Res Pract Thromb Haemost.

[10] de Kleijn R.J.C.M.F., Schropp L., van Hattum E.S., Ünlu Ç., Middeldorp S., Nijkeuter M. (2022). Post-thrombotic syndrome after upper extremity deep vein thrombosis: an international Delphi consensus study. J Thromb Haemost.

[11] Schulman S., Kearon C. (2005). Definition of major bleeding in clinical investigations of antihemostatic medicinal products in non-surgical patients. J Thromb Haemost.

[12] Kaatz S., Ahmad D., Spyropoulos A.C., Schulman S. (2015). Definition of clinically relevant non-major bleeding in studies of anticoagulants in atrial fibrillation and venous thromboembolic disease in non-surgical patients: communication from the SSC of the ISTH. J Thromb Haemost.

[13] Kahn S.R., Elman E.A., Bornais C., Blostein M., Wells P.S. (2005). Post-thrombotic syndrome, functional disability and quality of life after upper extremity deep venous thrombosis in adults. Thromb Haemost.

[14] Avila M.L., Duan L., Cipolla A., Kim A., Kahr W.H.A., Williams S., Brandão L.R. (2014). Postthrombotic syndrome following upper extremity deep vein thrombosis in children. Blood.

[15] Etxeandia-Ikobaltzeta I., Zhang Y., Brundisini F., Florez I.D., Wiercioch W., Nieuwlaat R. (2020). Patient values and preferences regarding VTE disease: a systematic review to inform American Society of Hematology guidelines. Blood Adv.

[16] Bleker S.M., van Es N., Kleinjan A., Büller H.R., Kamphuisen P.W., Aggarwal A. (2016). Current management strategies and long-term clinical outcomes of upper extremity venous thrombosis. J Thromb Haemost.

[17] Kearon C., Akl E.A., Comerota A.J., Prandoni P., Bounameaux H., Goldhaber S.Z. (2012). Antithrombotic therapy for VTE disease: Antithrombotic Therapy and Prevention of Thrombosis, 9th ed: American College of Chest Physicians Evidence-Based Clinical Practice Guidelines. Chest.

[18] Kucher N. (2011). Clinical practice. Deep-vein thrombosis of the upper extremities. N Engl J Med.

[19] Barco S., Jalaie H., Sebastian T., Wolf S., Fumagalli R.M., Lichtenberg M. (2025). Aspirin plus rivaroxaban versus rivaroxaban alone for the prevention of venous stent thrombosis among patients with post-thrombotic syndrome: the multicenter, multinational, randomized, open-label ARIVA trial. Circulation.

[20] Kakkos S.K., Gohel M., Baekgaard N., Bauersachs R., Bellmunt-Montoya S., Black S.A. (2021). Editor’s Choice—European Society for Vascular Surgery (ESVS) 2021 clinical practice guidelines on the management of venous thrombosis. Eur J Vasc Endovasc Surg.

[21] Steiner D., Nopp S., Hoberstorfer T., Pabinger I., Weber B., Ay C. (2024). Anxiety in patients with venous thromboembolism: quantification and risk factors in a prospective cohort study. J Thromb Haemost.

[22] Ruiz-Artacho P, Lecumberri R, Trujillo-Santos J, Beddar Chaib F, Moustafa F, Lorenzo A (2025). Direct oral anticoagulants versus standard therapy in upper extremity deep vein thrombosis: real-world evidence. Thromb Haemost.

[23] Espitia O., Raimbeau A., Planquette B., Katsahian S., Sanchez O., Espinasse B. (2024). A systematic review and meta-analysis of the incidence of post-thrombotic syndrome, recurrent thromboembolism, and bleeding after upper extremity vein thrombosis. J Vasc Surg Venous Lymphat Disord.

[24] Valeriani E., Di Nisio M., Porceddu E., Agostini F., Pola R., Spoto S. (2022). Anticoagulant treatment for upper extremity deep vein thrombosis: a systematic review and meta-analysis. J Thromb Haemost.

