# Supplementary material for: Patient-reported outcomes after upper-extremity deep vein thrombosis due to compression syndromes: a multinational longitudinal retrospective study
Source: Res Pract Thromb Haemost. 2026 Jan 16;10(1):103358. doi: 10.1016/j.rpth.2026.103358 (PMC12907070; doi:10.1016/j.rpth.2026.103358)
Supplement: Supplementary Material [file mmc1.docx]

**Supplementary Material**

**Supplementary Table S1. Median values of QuickDASH Score and adapted visual analogue scale across countries**

|  | | **Adapted VAS** | | | | | | | | | | **QuickDASH Score** | | | | | | | | | |  |  |
| --- | --- | --- | --- | --- | --- | --- | --- | --- | --- | --- | --- | --- | --- | --- | --- | --- | --- | --- | --- | --- | --- | --- | --- |
|  | | *Switzerland* | *France* | | | | *Italy* | *Turkey* | | | | *Switzerland* | | | *France* | | | *Italy* | *Turkey* | | |  |  |
| Valid |  | 40 |  | 11 |  | 27 | | |  | 3 |  | 41 |  | 11 | |  | 27 | | |  | 3 | |  |
| Missing |  | 1 |  | 0 |  | 0 | | |  | 0 |  | 0 |  | 0 | |  | 0 | | |  | 0 | |  |
| Median |  | 8.000 |  | 7.500 |  | 7.500 | | |  | 8.000 |  | 4.545 |  | 18.182 | |  | 13.636 | | |  | 9.091 | |  |
| 25th percentile |  | 7.750 |  | 7.000 |  | 6.000 | | |  | 7.500 |  | 0.000 |  | 12.500 | |  | 2.273 | | |  | 4.545 | |  |
| 75th percentile |  | 10.000 |  | 8.000 |  | 9.000 | | |  | 8.500 |  | 13.636 |  | 25.000 | |  | 25.000 | | |  | 10.227 | |  |

VAS: Visual Analogue Scale; QuickDASH: Quick Disabilities of the Arm, Shoulder, and Hand

**Supplementary Table S2. Symptom-Specific Predictors of QuickDASH Score: Multivariable Linear Regression Model**

QuickDASH: Quick Disabilities of the Arm, Shoulder, and Hand

|  | | |  | | | | | | | | | |  |  |  |
| --- | --- | --- | --- | --- | --- | --- | --- | --- | --- | --- | --- | --- | --- | --- | --- |
|  | | **Symptom** | | | **B Coefficient** | | **Standard Error** | | **t-value** | **p-value^1^** | | |  |  |  |
|  |  | Intercept | |  | 5.48 |  | 1.70 |  | 3.23 | 0.002 |  |  | | |  |
|  |  | Swelling | |  | 6.49 |  | 3.15 |  | 2.06 | 0.043 |  |  | | |  |
|  |  | Pain | |  | 0.52 |  | 4.06 |  | 0.13 | 0.898 |  |  | | |  |
|  |  | Heaviness | |  | 4.71 |  | 3.10 |  | 1.52 | 0.133 |  |  | | |  |
|  |  | Limited mobility | |  | 19.86 |  | 4.70 |  | 4.22 | < .001 |  |  | | |  |
|  |  | Skin discoloration | |  | 20.61 |  | 5.06 |  | 4.07 | < .001 |  |  | | |  |
|  |  | Paresthesia | |  | 6.71 |  | 4.52 |  | 1.48 | 0.142 |  |  | | |  |
|  |  | Weakness | |  | 17.78 |  | 7.11 |  | 2.50 | 0.015 |  |  | | |  |
|  |  | Discomfort | |  | 3.32 |  | 5.80 |  | 0.56 | 0.579 |  |  | | |  |
|  | | |  | | | | | | | | | | |  |  |
|  | ^1^Bold *p*-value indicates statistical significance (*p* < 0.05) | | | | | | | | | | | | |  |  |
|  | | |  | | | | | | | | | | |  |  |

**Supplementary Table S3. Association between individual QuickDASH items and the adapted visual analogue scale for quality of life**

|  | |  | |  | |  | | |  |  | | **95% CI** |  | |  |  |
| --- | --- | --- | --- | --- | --- | --- | --- | --- | --- | --- | --- | --- | --- | --- | --- | --- |
|  | | **QuickDash Items** | | **B Coefficient** | | **Standard Error** | | | **t-value** | **p-value^1^** | | **Lower** | **Upper** | |  |  |
|  |  | QuickDASH 1 | 0.50 | | |  | 0.15 |  | 3.40 | | **0.001** | 0.205 | | 0.798 | |  |
|  |  | QuickDASH 2 | -0.14 | | |  | 0.19 |  | -0.90 | | 0.373 | 0.562 | | 0.215 | |  |
|  |  | QuickDASH 3 | -0.20 | | |  | 0.16 |  | -1.30 | | 0.199 | 0.513 | | 0.110 | |  |
|  |  | QuickDASH 4 | 0.89 | |  | | 0.25 |  | 3.66 | | **< .001** | 0.402 | | 1.384 | |  |
|  |  | QuickDASH 5 | -0.94 | |  | | 0.24 |  | -3.91 | | **< .001** | 1.423 | | -0.456 | |  |
|  |  | QuickDASH 6 | -0.68 | |  | | 0.15 |  | -4.46 | | **< .001** | 0.984 | | -0.372 | |  |
|  |  | QuickDASH 7 | -0.96 | |  | | 0.22 |  | -4.29 | | **< .001** | 1.408 | | -0.509 | |  |
|  |  | QuickDASH 8 | 0.31 | |  | | 0.17 |  | 1.80 | | 0.079 | 0.037 | | 0.647 | |  |
|  |  | QuickDASH 9 | 0.12 | | |  | 0.22 |  | 0.52 | | 0.603 | 0.325 | | 0.554 | |  |
|  |  | QuickDASH 10 | -0.54 | | |  | 0.20 |  | -2.65 | | 0.011 | 0.945 | | -0.130 | |  |
|  |  | QuickDASH 11 | 0.40 | | |  | 0 |  | 1.62 | | 0.111 | -0.10 | | 0.090 | |  |
|  |  |  |  | | |  |  |  |  | |  |  | |  | |  |

^1^Bold *p*-values indicate statistical significance (*p* < 0.05).

QuickDASH: Quick Disabilities of the Arm, Shoulder, and Hand; CI: confidence interval

**Supplementary Table S4. Patient-defined treatment priorities, level of satisfaction, and perceived unmet needs after acute treatment**

|  | **N=82** |
| --- | --- |
| Patient-Identified Priorities for Treatment, n (%) |  |
| Maintaining arm function | 64 (78.0%) |
| Preventing thrombosis | 60 (73.0%) |
| Minimization of bleeding risks | 24 (29.0%) |
| Shortening treatment duration | 10 (12.0%) |
| Preventing hospital readmission | 17 (21.0%) |
| Least invasive treatment possible | 17 (21.0%) |
| Symptom relief (e.g. swelling, pain, heaviness) | 41 (50.0%) |
| Symptoms not sufficiently addressed during treatment, n (%) |  |
| Swelling of the arm | 5 (6.1%) |
| Pain | 6 (7.3%) |
| Heaviness or fatigue in the arm | 15 (18.0%) |
| Restricted movement of the arm or shoulder | 4 (4.9%) |
| Paresthesia | 8 (9.8%) |
| Impaired hand function | 2 (2.4%) |
| Other | 5 (6.1%) |
| I had no unnoticed symptoms | 45 (55.0%) |
| Felt like concerns were taken seriously by treatment team, n (%) |  |
| Yes, completely | 57 (70.0%) |
| Yes, mostly | 18 (22.0%) |
| Partially | 2 (2.5%) |
| No, rather not | 3 (3.7%) |
| No, not at all | 1 (1.2%) |
| How well was treatment tailored to individual needs, n (%) |  |
| Optimally tailored | 40 (49.0%) |
| Satisfactory | 32 (40.0%) |
| Neutral | 5 (6.2%) |
| Inadquate | 2 (2.5%) |
| No consideration | 1 (1.2%) |
| Not applicable | 1 (1.2%) |
| Suggested areas for improvement, n (%) |  |
| More detailed explanation of the diagnosis and treatment options | 9 (11.0%) |
| More time for questions and concerns | 12 (15.0%) |
| Better consideration of symptoms | 9 (11.0%) |
| More individualized treatment decision | 10 (12.0%) |
| Less invasive treatment options | 7 (8.5%) |
| More support in coping with the consequences of the condition | 30 (37%) |
| Other | 3 (3.7%) |
| None | 21 (26.0%) |
| Overall satisfaction with treatment, n (%) |  |
| Very satisfied | 38 (47.0%) |
| Satisfied | 36 (44.0%) |
| Neutral | 4 (4.9%) |
| Dissatisfied | 3 (3.7%) |
| Very dissatisfied | 0 |

**Supplementary Figure S1. Correlation plots depicting the median (Q1-Q3) adapted visual analogue scale for quality of life across the severity groups of the eleven QuickDASH items
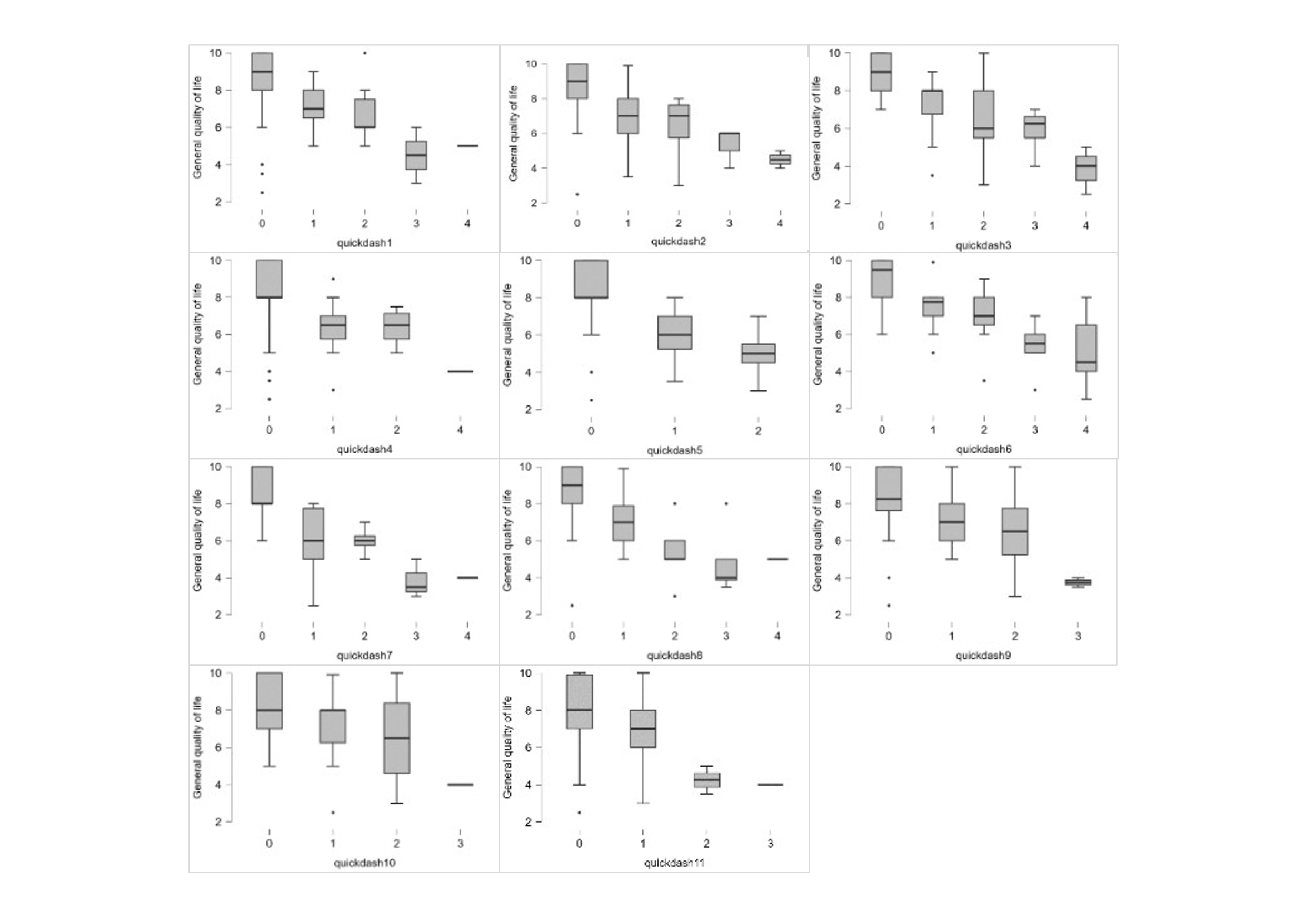
**

Boxplots illustrate the distribution of adapted visual analogue scale for quality of life according to patient-reported impairment in each QuickDASH subitem (0 = no impairment, 4 = maximum impairment).

QuickDASH: Quick Disabilities of the Arm, Shoulder, and Hand

**Supplementary Figure S2. Correlation plots depicting the distribution of QuickDASH score points by adapted visual analogue scale for quality of life**

| Switzerland  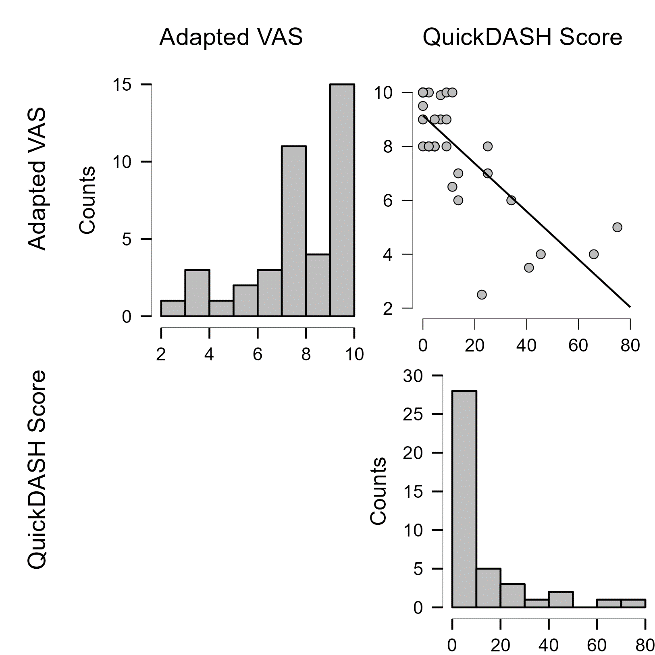 | France  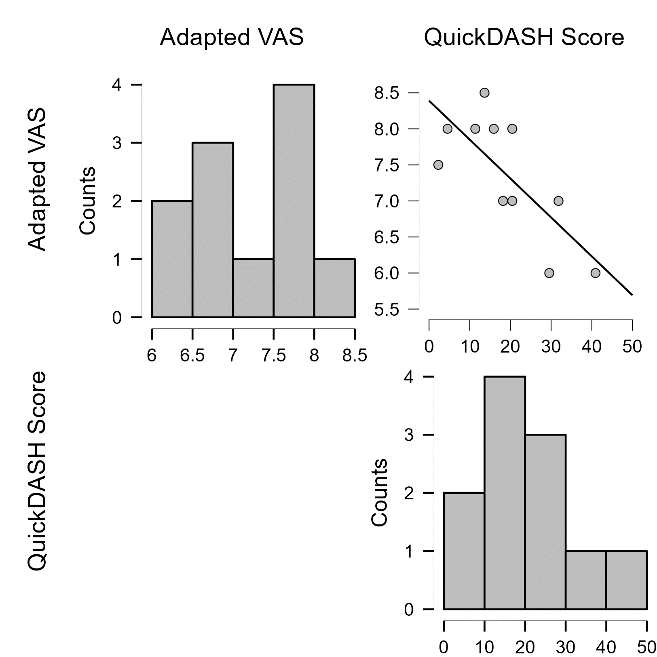 |
| --- | --- |
| Italy  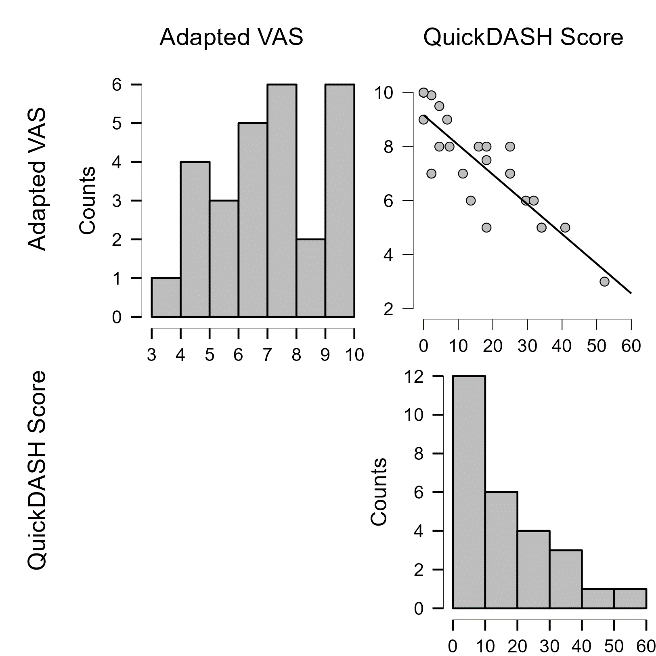 | Turkey  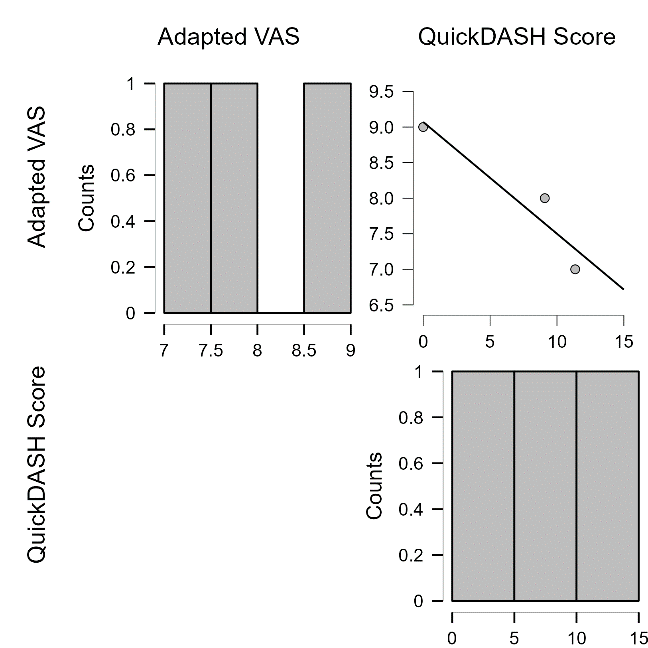 |

VAS: Visual Analogue Scale; QuickDASH: Quick Disabilities of the Arm, Shoulder, and Hand

**Supplementary Figure S3. QuickDASH score stratified by self-reported mental health impact**


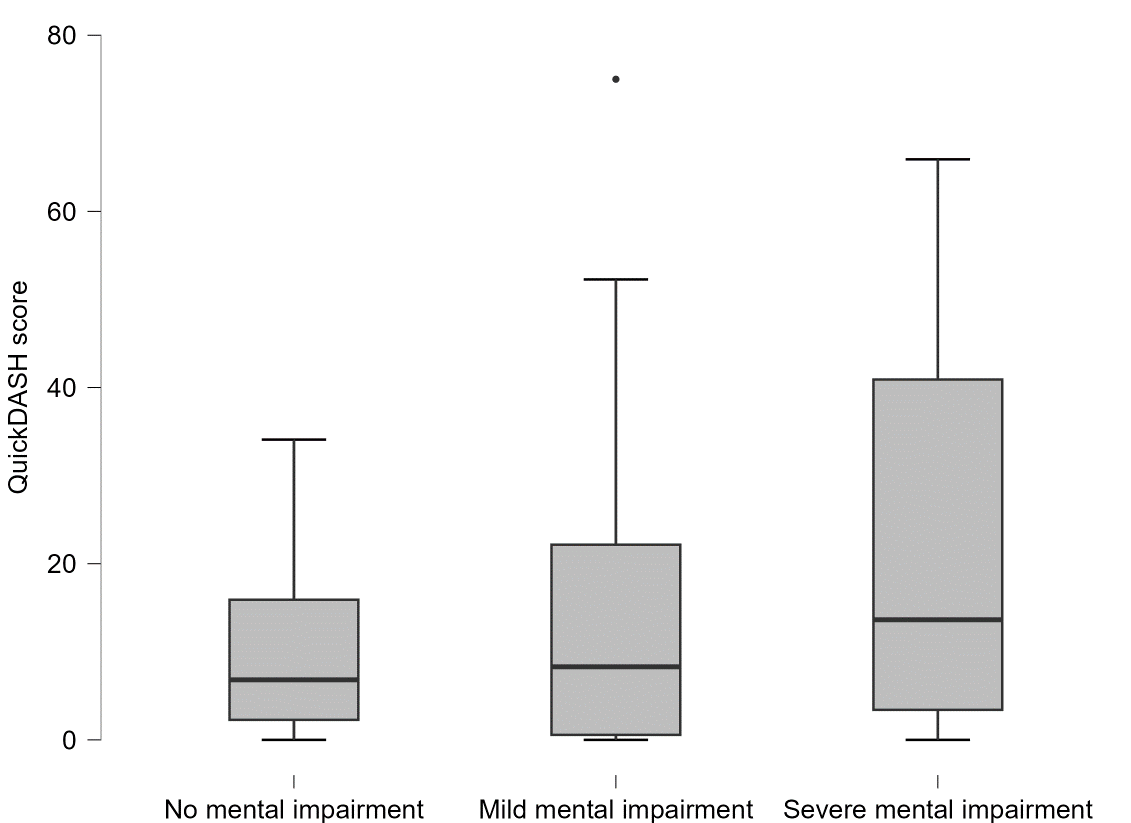


QuickDASH: Quick Disabilities of the Arm, Shoulder, and Hand

**Questionnaire**

| Information collected by physician before phone call with patient | | |
| --- | --- | --- |
| Baseline Characteristics | Variable Type/ Response Options | **Data Entry** |
| Patient identifier | Free text/ Numerical value  (i.e. ZUR1, ZUR2, …) | ___ ___ ___ ___ |
| Age at treatment (years) | Add a numeric value | ___ ___ |
| Sex at birth | \|_\| Male  \|_\| Female |  |
| BMI (kg/m^2^) | Numeric value | ___ ___ |
| Medical history | Cross on one or more (multiple answers possible)  \|_\| None  \|_\| Known cardiovascular diseases  \|_\| Known diabetes  \|_\| Known malignancy (active or under treatment)  \|_\| Dyslipidemia,  \|_\| Oral estrogenic contraception or pregnancy  \|_\| Prior VTE  \|_\| Family history of VTE  \|_\| Other (Please specify) | |
| Diagnosis | \|_\| Phenotype 1 = Effort thrombosis/ Paget-von-Schroetter syndrome in physically active patients, or  \|_\| Phenotype 2 = Secondary compression or other chronic disorder (operation, arthrosis, malignancy, infection, … ) not related to CVC or peripheral line | |
| Time of functional assessment since initial diagnosis (months) | Numeric value | ___ ___ |
| Thrombus location | One or more answers    \|_\| Anonyma or central vein (superior vena cava)  \|_\| Subclavian vein  \|_\| Axillary vein  \|_\| Brachial vein  \|_\| Forearm  \|_\| Superficial veins  Multiple answers possible |  |
| Thrombus length (cm) | Numeric value (approx.) | ___ ___ |
| Compression at thoracic outlet | \|_\| No  \|_\| Yes |  |
| Risk factors | \|_\| Anatomical narrowing  \|_\| Trauma or injury  \|_\| Repeated mechanical stress/ sport exposition  \|_\| Thrombophilia (FV Leiden mutation, prothrombin mutation, PC/PS/AT deficiency, APS)  \|_\| Long travel haul  \|_\| Iatrogenic (recent intervention, medication)  \|_\| Unprovoked  Multiple answers possible |  |
| Duration of anticoagulation (days) | Numeric value | ___ ___ |
| Use of compression stockings | \|_\| No  \|_\| Yes |  |
| Type of intervention | \|_\| None  \|_\| Catheter directed thrombolysis  \|_\| Percutaneous thrombectomy  \|_\| Balloon angioplasty  \|_\| Rib resection  \|_\| Thrombectomy  Multiple answers possible |  |
| Length of hospital stay, cumulative (days) | Numeric value | ___ ___ |
| Follow-up period (months) | Numeric value | ___ ___ |
| Recurrent thrombosis during follow-up | \|_\| No  \|_\| Yes |  |
| Major bleeding events during follow-up | \|_\| No  \|_\| Yes |  |

| Phone call with patient | | |
| --- | --- | --- |
| Patient-Reported Outcome Measures (Survey) | Variable Type/ Response Options | **Data Entry** |
| Are you currently experiencing symptoms in the affected arm? | \|_\| No  \|_\| Yes | |
| If yes, which symptoms are present? | \|_\| Swelling  \|_\| Pain  \|_\| Heaviness  \|_\| Restricted mobility  \|_\| Skin discoloration  \|_\| Visible veins  \|_\| Other  Multiple answers possible | |
| Are you currently taking any medication for the above-mentioned symptoms? | \|_\| No  \|_\| Yes | |
| Please rate your ability to do the following activities in the last week by circling the number below the appropriate response. | | |
| 1. Open a tight or new jar. (Ouvrez un bocal hermétique ou neuf.) (Apri un barattolo ermetico o nuovo.) | \|_\| No difficulty  \|_\| Mild difficulty  \|_\| Moderate difficulty  \|_\| Severe difficulty  \|_\| Unable | |
| 1. Do heavy household chores (e.g., wash floors) (Effectuer des tâches ménagères lourdes (par exemple, laver les sols)) (Eseguire lavori domestici pesanti (ad esempio lavare i pavimenti) | \|_\| No difficulty  \|_\| Mild difficulty  \|_\| Moderate difficulty  \|_\| Severe difficulty  \|_\| Unable | |
| 1. Carry a shopping bag or briefcase. | \|_\| No difficulty  \|_\| Mild difficulty  \|_\| Moderate difficulty  \|_\| Severe difficulty  \|_\| Unable | |
| 1. Wash your back. | \|_\| No difficulty  \|_\| Mild difficulty  \|_\| Moderate difficulty  \|_\| Severe difficulty  \|_\| Unable | |
| 1. Use a knife to cut food. | \|_\| No difficulty  \|_\| Mild difficulty  \|_\| Moderate difficulty  \|_\| Severe difficulty  \|_\| Unable | |
| 1. Recreational activities in which you take some force or impact through your arm, shoulder or hand (e.g., golf, hammering, tennis, etc.). | \|_\| No difficulty  \|_\| Mild difficulty  \|_\| Moderate difficulty  \|_\| Severe difficulty  \|_\| Unable | |
| During the past week, to what extent has your arm, shoulder or hand problem interfered with your normal social activities with family, friends, neighbours or groups? | \|_\| Not at all  \|_\| Slightly  \|_\| Moderately  \|_\| Quite a bit  \|_\| Extremely |  |
| During the past week, were you limited in your work or other regular daily activities as a result of your arm, shoulder or hand problem? | \|_\| Not limited at all  \|_\| Slightly limited  \|_\| Moderately limited  \|_\| Very limited  \|_\| Unable |  |
| Please rate the severity of the following symptoms in the last week. | -- |  |
| 1. Arm, shoulder or hand pain. | \|_\| None  \|_\| Mild  \|_\| Moderate  \|_\| Severe  \|_\| Extreme |  |
| 1. Tingling (pins and needles) in your arm, shoulder or hand. | \|_\| None  \|_\| Mild  \|_\| Moderate  \|_\| Severe  \|_\| Extreme |  |
| During the past week, how much difficulty have you had sleeping because of the pain in your arm, shoulder or hand? | \|_\| No difficulty  \|_\| Mild difficulty  \|_\| Moderate difficulty  \|_\| Severe difficulty  \|_\| So much difficulty that I can't sleep | |
| How would you rate your current quality of life compared to the time before your illness?  Worst quality of life imaginable = 0 points to best quality of life imaginable = 100 points (corresponding to before thrombosis) | Numeric value | **___ ___ ___** |
| Did you experience any bleeding during or after the treatment? | \|_\| No  \|_\| Yes |  |
| If yes, how was the most severe bleeding event classified? | \|_\| Minor (e.g. bruises, nosebleeds)  \|_\| Moderate (e.g. hospitalization required, transfusion necessary)  \|_\| Major (e.g. brain hemorrhage, life-threatening bleeding or intervention required) |  |
| Are you still receiving anticoagulation therapy? | \|_\| No  \|_\| Yes  \|_\| Yes, temporarily |  |
| Has your condition affected your mental health? | \|_\| No  \|_\| Yes, severely  \|_\| Yes, mildly |  |
| If yes, what effects have you noticed? | \|_\| Fear of (recurrent) thrombosis  \|_\| Fear of (recurrent) bleeding  \|_\| Restricted social activities  \|_\| Sleep problems  \|_\| Other |  |
| Have you ever received psychological support (e.g., counseling or therapy) due to your condition? | \|_\| No, but I would have liked it  \|_\| No, it wasn't necessary  \|_\| Yes |  |
| Which factors were most important to you during your treatment? | \|_\| Maintaining arm function  \|_\| Preventing thrombosis  \|_\| Minimization of bleeding risks  \|_\| Shortening treatment duration  \|_\| Preventing hospital readmission  \|_\| Least invasive treatment possible  \|_\| Symptom relief (e.g. swelling, pain, heaviness) | |
| Which of your symptoms do you think were not sufficiently addressed during treatment? | \|_\| Swelling of the arm  \|_\| Pain  \|_\| Heaviness or fatigue in the arm  \|_\| Restricted movement of the arm or shoulder  \|_\| Numbness or tingling  \|_\| Change in skin color or temperature of the arm  \|_\| Impaired hand function  \|_\| Other  \|_\| I had no unnoticed symptoms  Multiple answers possible | |
| Did you feel that your concerns were taken seriously by your doctor or treatment team | \|_\| Yes, completely  \|_\| Yes, mostly  \|_\| Partially  \|_\| No, rather not  \|_\| No, not at all |  |
| How well was your treatment tailored to your individual needs? | \|_\| Optimally tailored  \|_\| Satisfactory  \|_\| Neutral  \|_\| Inadequate  \|_\| No consideration |  |
| What did you feel was lacking or could have been better? | \|_\| More detailed explanation of the diagnosis and treatment options,  \|_\| More time for my questions and concerns  \|_\| Better consideration of my symptoms  \|_\| More individualized treatment decisions  \|_\| Less invasive treatment options  \|_\| More support in coping with the consequences of the condition  \|_\| Other  Multiple answers possible | |
| How satisfied are you with your treatment overall? | \|_\| Very satisfied  \|_\| Satisfied  \|_\| Neutral  \|_\| Dissatisfied  \|_\| Very dissatisfied |  |
| Do you have anything else you would like to tell us about your treatment or your experience with your condition? | Free text | |
